# Research status and progress of Chinese traditional medicine for chronic prostatitis/chronic pelvic pain syndrome: A bibliometric analysis and literature review

**DOI:** 10.1097/MD.0000000000047125

**Published:** 2026-01-16

**Authors:** Debo Li, Yongfeng Lao, Xin Guan, Xiangbin Kong, Zhilong Dong

**Affiliations:** a Department of Urology, The Second Hospital & Clinical Medical School, Lanzhou University, Lanzhou, Gansu, China; b Institute of Urology, Gansu Province Clinical Research Center for Urinary System Diseases, The Second Hospital of Lanzhou University, Lanzhou, Gansu, China.

**Keywords:** acupuncture, Chinese herbal formulas, chronic prostatitis/chronic pelvic pain syndrome (CP/CPPS), traditional Chinese medicine (TCM), Tuina massage

## Abstract

**Background:**

Chronic prostatitis/chronic pelvic pain syndrome (CP/CPPS) is a prevalent and complex condition in men that significantly impairs patients’ quality of life. While current Western medical treatments offer limited efficacy, traditional Chinese medicine (TCM), with its distinctive system of syndrome differentiation, provides a range of therapeutic approaches for CP/CPPS. These include acupuncture, herbal prescriptions, Tuina massage, and other complementary interventions, often delivered through an integrated treatment strategy. This approach not only demonstrates notable therapeutic effectiveness but also exhibits a high safety profile.

**Methods:**

This study integrates bibliometric analysis with a literature review. We retrieved publications on TCM for CP/CPPS from the Web of Science Core Collection (from its inception to April 2025). Analytical tools such as VOSviewer and CiteSpace were employed to examine publication trends, countries/institutions, and keywords, etc. Concurrently, the review synthesizes evidence on the efficacy and mechanisms of TCM interventions for CP/CPPS.

**Results:**

The study analyzed 106 publications from 19 countries (2003–2025), revealing a gradual increase in annual publication output in recent years. China contributed the most publications (79 papers), with leading research institutions including Beijing University of Chinese Medicine and China Academy of Chinese Medical Sciences. The journal Medicine was the most frequently published venue. In TCM theory, CP/CPPS is associated with dampness-heat, blood stasis, and qi stagnation, involving multiple meridians and organs, primarily the Bladder Meridian of Foot-Taiyang, etc. Acupuncture is identified as a core intervention. Potential mechanisms of TCM may include immunomodulation, enhanced local blood circulation, reduced inflammatory response, and regulation of the neuroendocrine system.

**Conclusion:**

Bibliometric analysis outlines the research trends and developments in TCM for CP/CPPS. The review synthesizes potential therapeutic mechanisms and efficacy of TCM, demonstrating that comprehensive TCM therapies exhibit well-documented efficacy and a favorable safety profile, thereby providing valuable references for clinical prevention and treatment of CP/CPPS. Future efforts should focus on further mechanistic exploration, promotion of high-quality research, and optimization of clinical protocols to facilitate broader application.

## 1. Introduction

Chronic prostatitis (CP) ranks among the most challenging clinical conditions encountered in urological practice. It is broadly categorized into 2 types: chronic bacterial prostatitis and chronic nonbacterial prostatitis (CNP), with the latter also commonly designated as CP/CPPS.^[1]^ According to the report, the incidence rate of CP has shown a significant upward trend, which has had a profound impact on the quality of life (QoL) of patients.^[2]^ Among all CP patients, CP/CPPS constitutes the overwhelming majority.^[3]^ Due to diverse clinical presentations and complex pathophysiology, CP still lacks standardized therapeutic protocols. Consequently, clinical practice is evolving toward multidisciplinary integrated approaches.^[4,5]^

Traditional Chinese medicine (TCM) constitutes an invaluable heritage of the Chinese nation and occupies a pivotal position within China’s healthcare system. Characterized by holistic treatment principles, personalized therapeutic strategies, and empirically rich modalities, TCM encompasses diverse interventions such as herbal medicine, acupuncture, and Tuina massage. TCM has been extensively utilized for systematic management of complex diseases and disorders.^[6]^ TCM is now extensively applied in treating CP. A growing body of research is being published to advance the clinical implementation and mechanistic elucidation of TCM for CP management. This might be one of the popular directions for the clinical treatment of prostatitis. However, despite the growing volume of research, significant fragmentation persists within this field. What constitutes the most effective TCM therapeutic regimen? What are its underlying mechanisms of action? And what directions should future research prioritize? This study therefore aims to address these critical questions. Collectively, to comprehensively summarize research advances and trends regarding TCM applications and therapeutic mechanisms for CP, this study employed a combined approach integrating bibliometric analysis with narrative literature review.

Bibliometric analysis is a widely adopted literature review approach for analyzing and identifying the development status and research patterns within specific academic domains.^[7]^ Bibliometric analysis enables objective extraction of quantitative and qualitative characteristics (such as publication volume, citation counts, research hotspots, and collaborative networks) within specific research domains. This approach mitigates subjectivity in assessment and establishes a relatively objective evaluation methodology.^[8]^ Chen et al and Wang et al evaluated the current status and trends in prostatitis and urological pelvic pain syndromes research, respectively, and summarized key research hotspots and future directions.^[9,10]^ However, to our knowledge, no bibliometric analysis has been published specifically addressing TCM applications in CP. Consequently, this study conducts the 1st bibliometric analysis to summarize the research landscape and trends of TCM for CP management. Additionally, this study employs a narrative literature review to systematically examine TCM-focused CP from a granular perspective, including pharmacological mechanisms and therapeutic efficacy. In essence, by integrating the strengths of both methodologies, this research synthesizes advancements in TCM for CP management, thereby providing enhanced insights into the current landscape and evolving trends of TCM-based CP research.

## 2. Bibliometric analysis

### 2.1. Literature search and data analysis

To perform the bibliometric analysis, we conducted a systematic literature search to identify publications related to TCM therapy for CP in the Web of Science Core Collection (WOSCC), with a retrieval period spanning from the database’s inception through April 30, 2025.^[11,12]^ The following terms were used: ((((((((((((((((((((TS = (Chinese medicine)) OR TS = (traditional Chinese medicine)) OR TS = (Chinese traditional medicine)) OR TS = (Chinese patent medicine)) OR TS = (Chinese medicinal plant*)) OR TS = (Chinese herb*)) OR TS = (Chinese herbal medicine)) OR TS = (Chinese medicinal herb*)) OR TS = (Chinese herbology)) OR TS = (Chinese herbal formula*)) OR TS = (Chinese herbal compound*)) OR TS = (Chinese herbal prescription*)) OR TS = (Chinese herbal ingredient*)) OR TS = (Chinese herbal preparation*)) OR TS = (Chinese medicinal formula*)) OR TS = (Chinese decoction*)) OR TS = (Chinese formula*)) OR TS = (Chinese prescription*)) OR TS = (acupuncture)) OR TS = (moxibustion)) AND (((((((TS = (CP/CPPS)) OR TS = (CP‐CPPS)) OR TS = (chronic prostatitis/chronic pelvic pain syndrome)) OR TS = (chronic prostatitis with chronic pelvic pain syndrome)) OR TS = (chronic prostatitis and chronic pelvic pain syndrome)) OR TS = (chronic abacterial prostatitis)) OR TS = (chronic nonbacterial prostatitis)).

Only articles and reviews published in English were included. The retrieved records from WoSCC were exported in plain text format for subsequent bibliometric analysis. The following bibliometric indicators were analyzed: countries/regions, institutions, authors, journals, and keywords. Publication volume and citation counts serve as metrics for evaluating research productivity and impact, respectively.^[13,14]^ The exported records in plain text format were imported into VOSviewer (version 1.6.20) and CiteSpace (version 6.3.R1) for subsequent analysis. VOSviewer was employed to construct and visualize bibliometric networks encompassing countries/regions, institutions, authors, journals, and keywords. CiteSpace (version 6.3.R1) was employed to explore keyword clustering and burst detection. The data were subsequently imported into Microsoft Excel 2016 for generating line charts. Scimago Graphica (version 1.0.36) was also utilized for visualization where applicable.

### 2.2. Results of bibliometric analysis

#### 2.2.1. Trend analysis in publications and citations

**Figure 1.**
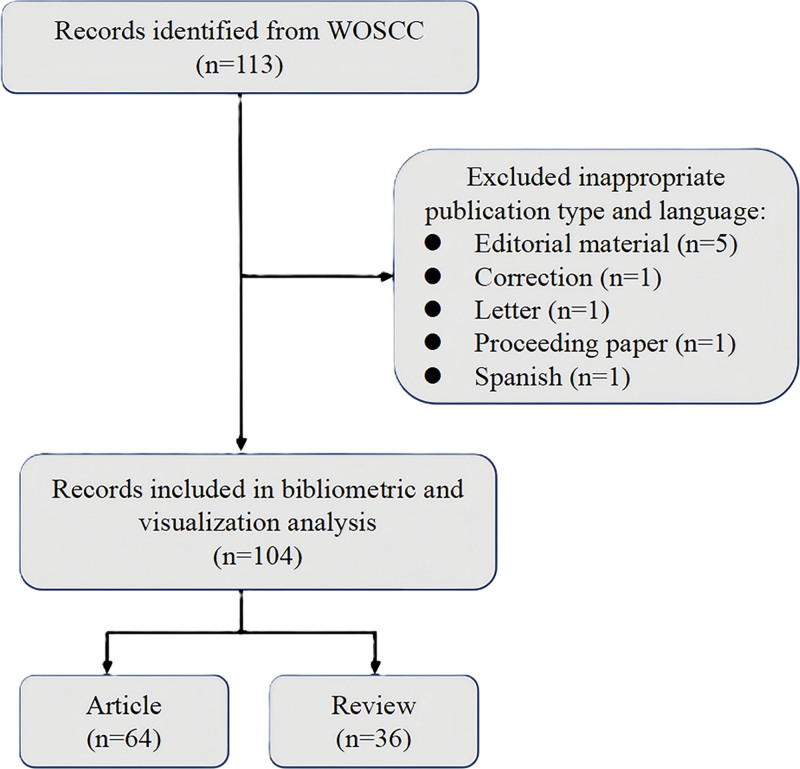
Flowchart of the search strategy and selection process.

**Figure 2.**
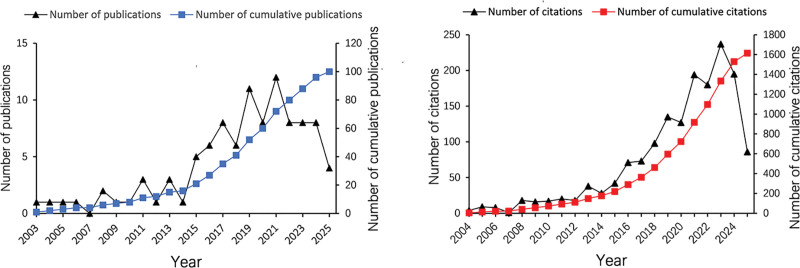
Publication volume and cumulative publication growth trends, along with citation counts and cumulative citation growth trends.

The initial search identified 114 records. After screening, 106 publications (68 articles and 38 reviews) published between 2003 and 2025 were included in the bibliometric analysis. The detailed screening procedure is presented in Figure 1. These 106 publications have collectively generated 1643 citations, averaging 15.5 citations per publication. The collective *H*-index for all records is 25, where the *H*-index is a widely adopted metric for assessing research productivity and impact. As shown in Figure 2, both annual publication volume and citation counts exhibit a general increasing trend despite year-to-year fluctuations. The year 2021 saw the highest number of papers published (12), after which the publication output began to decline and has remained at a relatively stable level in recent years. No publications were recorded in 2007, while 2023 demonstrated the peak citation count.

#### 2.2.2. Bibliometric analysis by country/region

**Figure 3.**
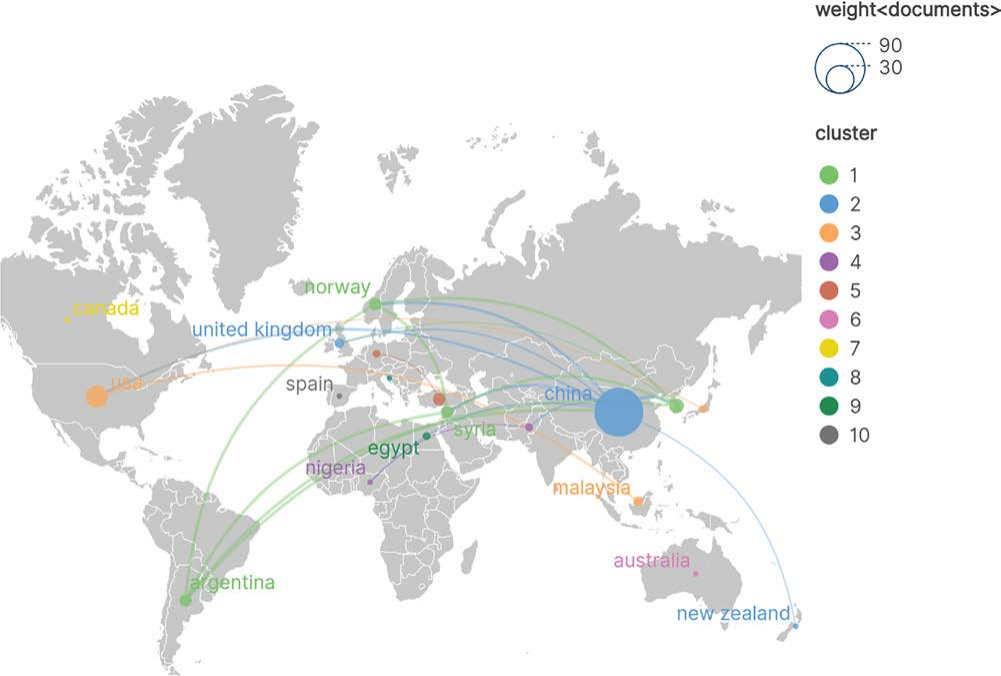
Country/region visualization. Node and edge sizes are weighted by publication volume. Node colors denote distinct clusters. Generated using Scimago Graphica (version 1.0.36).

From 2003 to 2025, 19 countries/regions contributed publications in this field. The top 3 countries/regions by publication volume were China (79 publications), the United States (16 publications), and South Korea (7 publications). The top 5 countries/regions by number of published papers are listed in Table 1. The global collaboration network in this field is shown in Figure 3. China makes the largest contribution and significantly outperforms other countries; however, it exhibits relatively weak collaboration ties with other nations.

**Table 1:** Top 5 productive countries/regions in traditional Chinese medicine and prostatitis.

Rank	Country	Record count	Citations	Average citation/publication
1	China	79	964	12.20
2	USA	16	483	30.19
3	South Korea	7	301	43.00
4	Argentina	5	198	39.60
5	Norway	5	198	39.60

#### 2.2.3. Bibliometric analysis by institution, journal, and author

**Figure 4.**
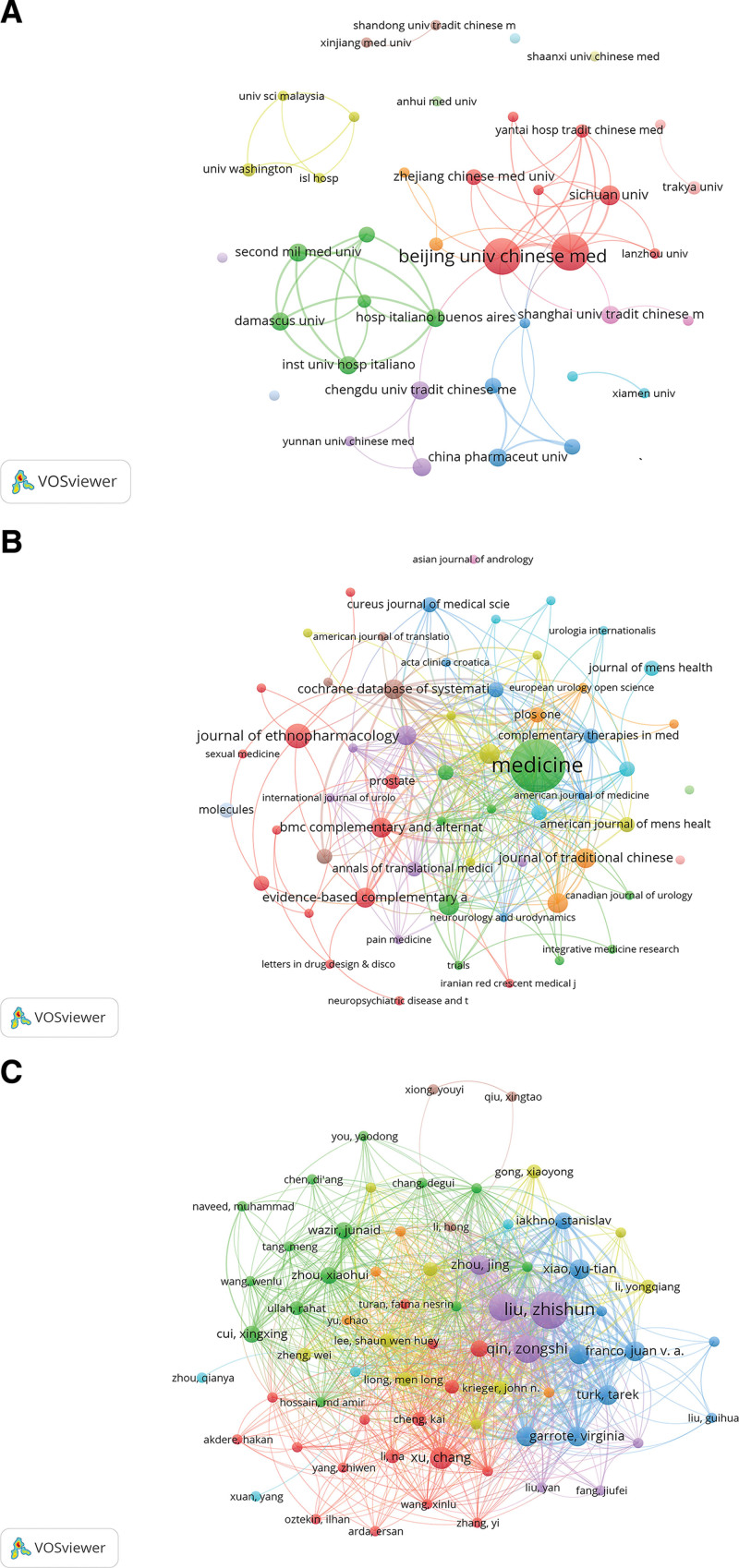
Global Institutional Collaboration Network (A); Journal Collaboration Network (B); Author Collaboration Network (C). Node and edge sizes are weighted by publication volume. Node colors denote distinct clusters.

Furthermore, 165 institutions have contributed publications. Beijing University of Chinese Medicine and China Academy of Chinese Medical Sciences both led in publication output (16 publications each), followed by Sichuan University (6 publications), Damascus University (5 publications), and Hospital Italiano de Buenos Aires (5 publications). The top 5 institutions by publication output are listed in Table 2, and the global collaboration network among institutions in this field is shown in Figure 4A. The Chinese Academy of Chinese Medical Sciences leads the field in both publication volume and citation count. The publications were disseminated across 60 journals. The top 10 journals by publication volume are presented in Table 3, with the top 5 being: Medicine (n = 12), Journal of Ethnopharmacology (n = 4), Urology (n = 3), Cochrane Database of Systematic Reviews (n = 3), and International Urology and Nephrology (n = 3). Journal collaboration networks are depicted in Figure 4B. Furthermore, 528 authors contributed to these publications. As presented in Table 4, the top 10 most productive authors are listed. The top 5 most prolific authors are all affiliated with institutions in China, while the 6th-ranked author is affiliated with Argentina. However, the Argentine author demonstrated higher citation counts than the top 5 Chinese authors. Author collaboration networks are visualized in Figure 4C. Finally, the top 10 most frequently cited publications are presented in Table 5, collectively cited 617 times (37.55% of total citations). Among these top-cited publications, 5 are review articles, 5 are original research articles, and 6 focus on acupuncture therapy for CP/CPPS.^[15–20]^ This indicates that acupuncture is a highly influential intervention method in the TCM treatment of CP/CPPS.

**Table 2:** Top 5 institutions in traditional Chinese medicine and prostatitis.

Rank	Institution	Record count	Citations	Total link strength
1	Chinese Academy of Chinese Medical Sciences	16	255	26
2	Beijing University of Chinese Medicine	16	241	25
3	Sichuan University	6	131	13
4	Damascus University	5	198	22
5	Hospital Italiano de Buenos Aires	5	198	22

**Table 3:** Top 10 journals in traditional Chinese medicine and prostatitis.

Rank	Source	Record count	JCR	IF
1	Medicine	12	Q4	1.4
2	Journal of Ethnopharmacology	4	Q2	5.4
3	Urology	3	Q3	2.0
4	Cochrane Database of Systematic Reviews	3	Q2	9.4
5	International Urology and Nephrology	3	Q4	1.9
6	Pain Research & Management	3	Q2	3.0
7	BMC Complementary and Alternative Medicine	3	Q1	3.4
8	Translational Andrology and Urology	3	Q3	1.9
9	Evidence-Based Complementary and Alternative Medicine	3	Q3	NA
10	Journal of Traditional Chinese Medicine	3	Q2	2.2

**Table 4:** Top 10 authors in traditional Chinese medicine and prostatitis.

Rank	Author	Record count	Citations	Average citation/publication	Total link strength
1	Liu, Zhishun	12	157	13.08	348
2	Qin, Zongshi	8	162	20.25	314
3	Wu, Jiani	8	162	20.25	314
4	Xu, Chang	6	61	10.17	124
5	Zhou, Jing	5	119	23.80	222
6	Franco, Juan V.A.	5	198	39.60	145
7	Garrote, Virginia	5	198	39.60	145
8	Jung, Jae Hung	5	198	39.60	145
9	Turk, Tarek	5	198	39.60	145
10	Vietto, Valeria	5	198	39.60	145

**Table 5:** Top 10 cited references in traditional Chinese medicine and prostatitis.

Rank	Cited reference	Citations	Total link strength
1	krieger jn, 1999, jama j am med assoc, v282, p236, doi 10.1001/jama.282.3.236	48	168
2	reesj, 2015, bju int, v116, p509, doi 10.1111/bju.13101	36	164
3	lee swh, 2008, am j med, v121, doi 10.1016/j.amjmed.2007.07.033	34	177
4	sahin s, 2015, prostate cancer p d, v18, p249, doi 10.1038/pcan.2015.13	33	178
5	lee sh, 2009, urology, v73, p1036, doi 10.1016/j.urology.2008.10.047	33	163
6	litwin ms, 1999, jurology, v162, p369, doi 10.1016/s0022-5347(05)68562-x	29	139
7	qin zs, 2018, j urology, v200, p815, doi 10.1016/jjure.2018.05.001	22	107
8	alexander rb, 2004, ann intern med, v141, p581, doi 10.7326/0003-4819-141-8-20.	20	99
9	chen r, 2003, urology, v61, p1156, doi 10.1016/s0090-4295(03)00141-9	20	79
10	lee swh, 2014, complement ther med, v22, p965, doi 10.1016/j.ctim.2014.10.010	19	110

#### 2.2.4. Bibliometric analysis of keywords

**Figure 5.**
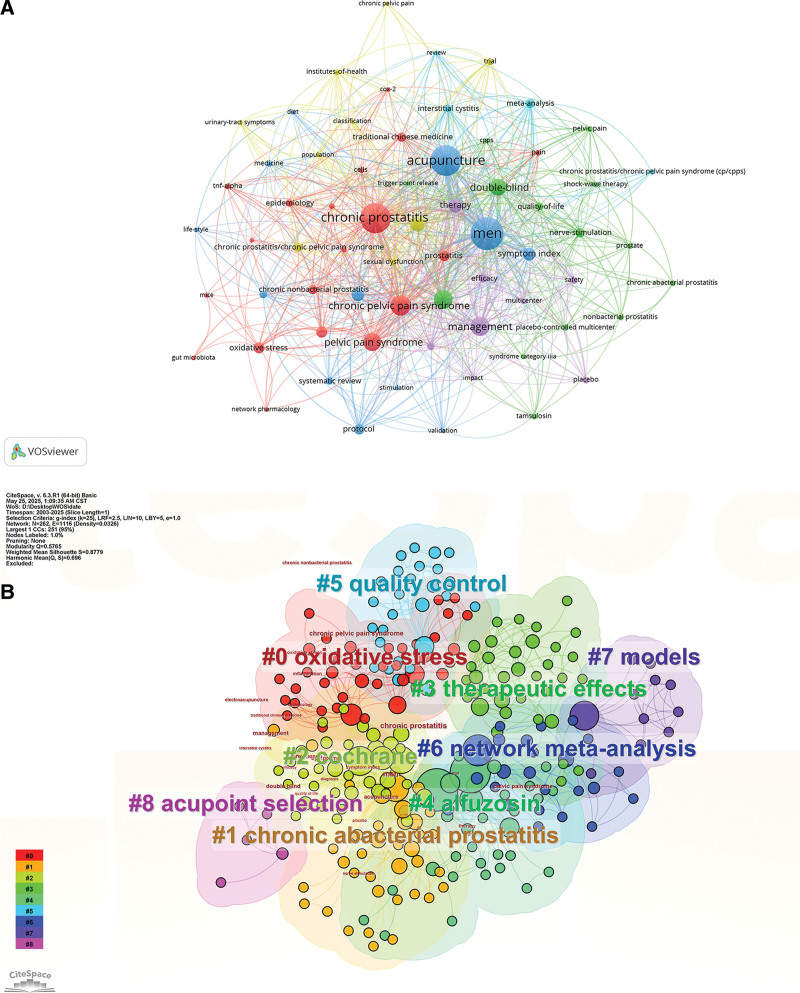
Keyword co-occurrence network (A) and keyword clustering analysis (B). Node and edge sizes are weighted by publication volume. Node colors denote distinct clusters. All of the keywords could be classified into 9 categories, which were oxidative stress, chronic abacterial prostatitis, cochrane, therapeutic effects, alfuzosin, quality control, network meta-analysis, models, and acupoint selection.

**Figure 6.**
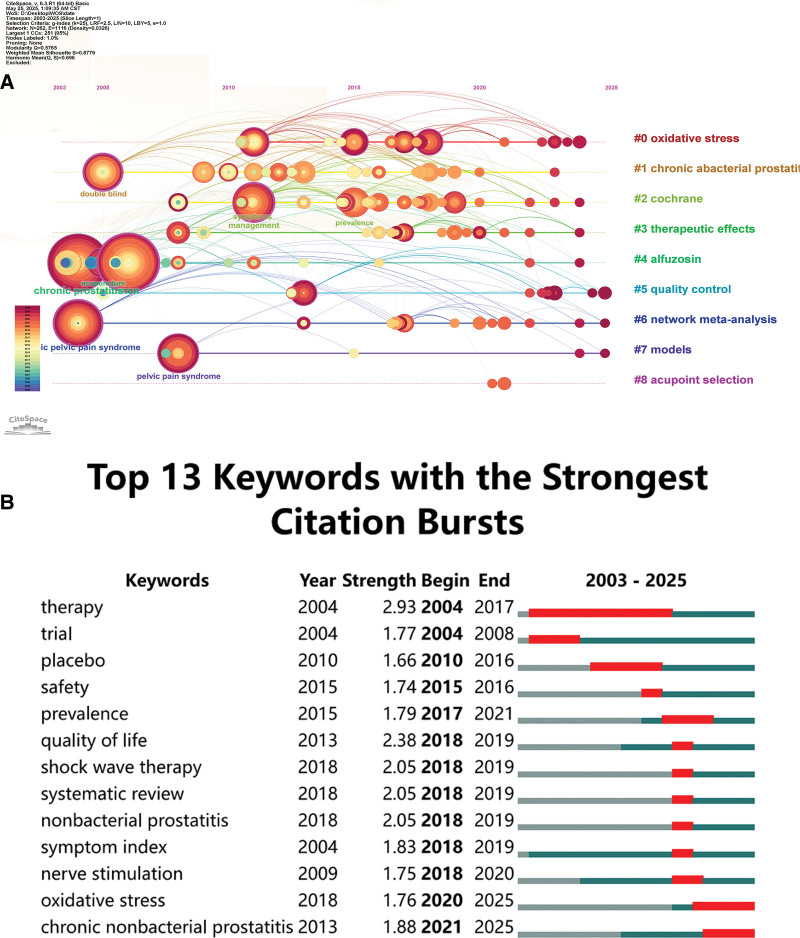
The timeline viewer of keywords (A). Top 13 keywords with the strongest citation bursts (B). The years between “beginning” and “end” represent periods when keywords were more influential. Light green years indicate the absence of keyword activity, deep green signifies low-intensity burst periods, and red denotes high-intensity burst activity.

Subsequently, we analyzed keyword frequencies, with the top 10 most frequent keywords listed in Table 6: Men (n = 48), Chronic Prostatitis (n = 43), Acupuncture (n = 43), Chronic Pelvic Pain Syndrome (n = 24), Symptoms (n = 21), Management (n = 21), Pelvic Pain Syndrome (n = 19), Double-Blind (n = 17), Therapy (n = 14), and Prevalence (n = 14). The keyword visualization is presented in Figure 5A. Subsequently, keyword clustering analysis was performed using CiteSpace, as shown in Figure 5B; (modularity *Q*-value = 0.5046, silhouette *S*-value = 0.7688). The results indicate that all keywords were grouped into 9 distinct clusters. The clusters were identified as: oxidative stress, chronic abacterial prostatitis, cochrane, therapeutic effects, alfuzosin, quality control, network meta-analysis, models, and acupoint selection. Additionally, we generated a timeline visualization map as shown in Figure 6A. Subsequently, we analyzed all data from 2003 to 2025 and identified burst keywords using CiteSpace. Burst keywords refer to terms exhibiting the sharpest frequency increases within specific time periods. This analysis highlights evolving research trends and hotspots across different timeframes while providing insights into future developmental directions. Figure 6B presents the top 13 keywords with the highest burst strength. Research in this domain has evolved, transitioning from prior emphasis on treatment modalities and outcome assessment to a current focus on mechanistic investigations. Oxidative stress and CNP emerged as the most prominent keywords over the past 5 years, exhibiting burst strengths of 1.76 and 1.88, respectively. While these values remain below 2.0. Notably, the relationship between oxidative stress and CNP is poised to emerge as a significant area of future research.

**Table 6:** Top 10 keywords in traditional Chinese medicine and prostatitis.

Rank	Keywords	Occurrences	Total link strength
1	Men	48	272
2	Chronic Prostatitis	43	223
3	Acupuncture	43	233
4	Chronic Pelvic Pain Syndrome	24	135
5	Symptoms	21	142
6	Management	21	132
7	Pelvic Pain Syndrome	19	79
8	Double-Blind	17	97
9	Therapy	14	57
10	Prevalence	14	105

## 3. Literature review on promising TCM treatment

Bibliometric findings indicate a growing body of research on TCM for the treatment of CP/CPPS. The research focus has gradually shifted from interventional approaches to mechanistic investigations, with numerous interventions proposed as potentially effective. In the following section, we will summarize the current state of research on the management of CP/CPPS, categorized by different interventional strategies.

### 3.1. Acupuncture

Acupuncture represents one of the most frequently employed TCM interventions for CP. Researchers have maintained a long-standing focus on acupuncture’s therapeutic efficacy for CP. As early as 2003, Chen et al demonstrated that acupuncture ameliorates symptoms and enhances quality of life in male patients with CP/CPPS. Numerous subsequent clinical trials have validated the therapeutic efficacy of acupuncture for CP.^[21,22]^ Lee et al examined the therapeutic efficacy of electroacupuncture for CP/CPPS. Their results demonstrated significant alleviation of pain symptoms through electroacupuncture. Furthermore, preliminary findings suggest that electroacupuncture modulates the expression of pro-inflammatory factors and cyclooxygenase-2, thereby reducing prostaglandin E2 (PGE2) levels. Then, in a randomized controlled trial (RCT), Kucuk et al found that acupuncture was more effective than pharmacotherapy (levofloxacin and ibuprofen) in relieving symptoms associated with prostatitis.^[23]^ Zhou et al compared the clinical efficacy of 2 types of acupuncture (long-needle acupuncture vs traditional acupuncture) in the treatment of CP/CPPS. The results indicated that the clinical efficacy of long-needle acupuncture in treating CP/CPPS was superior to that of traditional acupuncture.^[24]^ Additionally, a specific type of acupuncture, trigger point injection, was also considered for CP/CPPS. The authors found that trigger point injection, as an adjunct to physical therapy, was well-tolerated and resulted in symptom improvement in approximately half of the CP/CPPS patients, although its durability and long-term outcomes have not yet been validated.^[25]^ Furthermore, Honjo et al investigated the efficacy of acupuncture in treating CP with intrarenal pelvic venous congestion. They demonstrated that acupuncture not only alleviated symptoms associated with prostatitis but also improved intrarenal pelvic venous congestion.^[26]^ Given the observed inconsistencies in measurement outcomes, Zhou et al proposed to evaluate the clinical efficacy of acupuncture through a multimodal assessment protocol applied post-treatment.^[27]^ Additionally, Lee et al developed a minimally invasive acupuncture technique serving as an effective sham control to better evaluate the therapeutic efficacy of acupuncture for CP/CPPS.^[28]^ Furthermore, Lee et al developed a minimally invasive acupuncture methodology designed to function as an efficacious sham control, thereby enhancing the evaluation of acupuncture’s therapeutic efficacy for CP/CPPS. Current evidence suggests a potential dose–response relationship between acupuncture treatment sessions and therapeutic outcomes for CP/CPPS. Increased treatment frequency correlates with greater reduction in NIH Chronic Prostatitis Symptom Index (NIH-CPSI) scores, with 6 sessions potentially representing the minimum threshold for achieving clinically meaningful outcomes.^[29]^ Ming et al compared the clinical efficacy of meridian-sensation acupuncture versus non-meridian-sensation acupuncture for CP/CPPS. The results indicated that both acupuncture modalities alleviated clinical symptoms in CP/CPPS patients, while meridian-sensation acupuncture demonstrated superior therapeutic outcomes.^[30]^ Beyond standalone acupuncture therapy, Wang et al investigated an integrated clinical protocol combining acupuncture with conventional pharmacotherapy (including tamsulosin) for CP management.^[31]^ Subsequently, Lei et al proposed an acupuncture–Qianlie’an suppository integrated protocol to assess the clinical efficacy of acupuncture in CP management.^[32]^

Based on current research, numerous meta-analyses (MAs) have been conducted. Hempen and Hummelsberger reviewed and organized 862 systematic reviews/meta-analyses (SRs/MAs) from 2017 to 2022, which indicated that CP/CPPS falls into the category of “Clear Positive Evidence,” primarily for alleviating pain and improving quality of life in patients.^[33]^ Regarding acupuncture versus sham acupuncture, Qin et al included 12 RCTs involving a total of 1188 patients. The results demonstrated that the acupuncture group had significantly lower total NIH-CPSI scores compared to the sham acupuncture group, with a particularly notable reduction in the pain domain. Moderate improvements were also observed in the NIH-CPSI urinary domain and quality of life domain scores.^[34]^ Subsequently, Pan et al further validated these findings by incorporating 10 high-quality RCTs (Jadad score ≥ 4). The meta-analysis showed that acupuncture resulted in a reduction of 6.41 points (95% CI [−7.53, −5.29]) in the total NIH-CPSI score compared to sham acupuncture, with low heterogeneity (*I*² = 0%). Acupuncture was also associated with a decrease in International Prostate Symptom Scores, indicating improvement in lower urinary tract obstructive symptoms. Furthermore, only mild adverse events such as hematoma and pain were reported, demonstrating a favorable safety profile.^[35]^ In studies comparing acupuncture with pharmacological therapy, Li et al found that combining acupuncture with TCM (such as resolving blood stasis) can further enhance therapeutic efficacy. The treatment resulted in a reduction of 4.0 points in the total NIH-CPSI score, along with significant improvements in pain symptoms, voiding symptoms, and quality of life.^[36]^ A recent MA by Fang et al, which incorporated 20 studies, demonstrated that the acupuncture group exhibited superior overall effectiveness compared to the pharmacotherapy group (tamsulosin or levofloxacin), along with a significant reduction in NIH-CPSI scores.^[37]^ Regarding time-dependent therapeutic effects, Fang et al conducted a network MA comparing acupuncture and extracorporeal shock wave therapy, which revealed that extracorporeal shock wave therapy was superior to acupuncture in improving both the total NIH-CPSI score and pain subscore in the short term (<4 weeks) and medium term (8–12 weeks). However, no significant difference was observed between the 2 interventions in the long term (>24 weeks). Moreover, acupuncture exhibited greater advantages in long-term improvement in the quality of life domain. The long-term follow-up study by Qin et al also supports this conclusion, indicating that symptom relief following acupuncture treatment can persist for more than 6 months without a significant trend of recurrence.^[38]^ A summary of the included MAs is provided in Table 7.^[39–41]^

**Table 7:** MA of acupuncture treatment for CP/CPPS.

Author/year	Participants	Intervention	Comparison	Outcome	Conclusion
Hempen and Hummelsberger^[33]^	The 862 SRs/MAs encompass a substantial number of patients with CP/CPPS.	Acupuncture (including manual acupuncture, electroacupuncture, etc).	Sham acupuncture, pharmacotherapy (e.g., Alpha-Blockers), and no treatment.	1. Acupuncture demonstrates a “clear positive effect” in the treatment of CP/CPPS, based on the current evidence grading. 2. Compared to the period prior to 2017, the quality of evidence has significantly improved.	Acupuncture is an effective intervention for CP/CPPS, supported by good-quality evidence.
Qin et al^[34]^	A total of 12 RCTs comprising 1188 patients with CP/CPPS were included.	1. Acupuncture (manual/electroacupuncture). 2. Acupuncture combined with pharmacotherapy (e.g., levofloxacin).	1. Sham acupuncture. 2. Pharmacotherapy alone (e.g., tamsulosin, levofloxacin).	1. Acupuncture vs sham acupuncture: significantly reduced total NIH-CPSI scores and pain sub-scores. 2. Acupuncture combined pharmacotherapy vs. pharmacotherapy alone: demonstrated superior outcomes in total NIH-CPSI scores. 3. Adverse events: only mild subcutaneous hematoma was reported, with no severe events observed.	Acupuncture is effective in treating CP/CPPS, particularly in alleviating pain, with a favorable safety profile.
Pan et al^[35]^	A total of 10 RCTs with Jadad scores ≥ 4, comprising 798 patients with CP/CPPS, were included.	Acupuncture (manual/electroacupuncture)	1. Sham acupuncture 2. Pharmacotherapy alone (e.g., tamsulosin, ibuprofen)	1. Acupuncture vs sham acupuncture: significant reductions were observed in the total NIH-CPSI score and pain subscore; 2. Acupuncture vs pharmacotherapy: demonstrated a higher response rate with fewer adverse events.	The efficacy and safety of acupuncture for CP/CPPS are well-supported by high-quality evidence.
Li et al^[36]^	A total of 19 RCTs, comprising 1831 patients with CP/CPPS, were included.	Acupuncture (manual/electroacupuncture) combined with Chinese herbal medicine (e.g., heat-clearing and dampness-draining formulas/blood-activating and stasis-resolving formulas)	1. Acupuncture alone. 2. Chinese herbal medicine alone. 3. Pharmacotherapy alone	1. Combination therapy vs control group: increased overall response rate and significant reduction in total NIH-CPSI score were observed. 2. Reduction in TCM syndrome score was demonstrated without an increase in adverse events.	Acupuncture combined with Chinese herbal medicine is a safe and effective treatment for CP/CPPS, demonstrating superior outcomes compared to monotherapy.
Fang et al^[37]^	A total of 20 RCTs, comprising 1661 patients with CP/CPPS, were included.	Acupuncture (manual/electroacupuncture/scalp acupuncture)	1. Sham acupuncture 2. Pharmacotherapy alone (e.g., tamsulosin, antibiotics)	1. Acupuncture vs control: significant reduction in total NIH-CPSI score and significantly higher overall response rate were observed. 2. Significant improvement in pain subscore was demonstrated, while no statistically significant difference was found in the urinary domain score.	Acupuncture is more effective than sham acupuncture or pharmacotherapy alone for CP/CPPS.
Qin et al^[38]^	A total of 6 studies, comprising 310 patients with CP/CPPS, were included.	Acupuncture (manual/electroacupuncture)	Single-arm MA without parallel control groups	1. Follow-up exceeding 6 months: a response rate of 68.4% was observed, with a reduction of 14.8 points in the total NIH-CPSI score; 2. Continuous improvements were demonstrated in both pain and quality of life scores throughout the follow-up period.	Acupuncture demonstrates sustained long-term therapeutic efficacy for CP/CPPS.
Kang et al^[39]^	A total of 9 RCTs, comprising 525 patients with CP/CPPS, were included.	Acupuncture (manual/electroacupuncture)	1. Sham acupuncture 2. ESWT	1. Short-term (<4 weeks) and medium term (8–12 weeks): ESWT was superior to acupuncture in improving the total NIH-CPSI score. 2. Long-term (>24 weeks): No significant difference was observed between acupuncture and ESWT in therapeutic efficacy. 3. Safety: The incidence of adverse events was comparable between the 2 groups.	Acupuncture demonstrates favorable long-term efficacy in the treatment of CP/CPPS, though its short-term effectiveness is slightly inferior to that of ESWT.
Zhang et al^[40]^	A total of 10 RCTs, comprising 770 patients with CP/CPPS, were included.	1. Acupuncture (manual/electroacupuncture). 2. Acupuncture combined with pharmacotherapy (e.g., levofloxacin, tamsulosin)	1. Sham acupuncture 2. Pharmacotherapy alone (e.g., tamsulosin, levofloxacin)	1. Acupuncture vs sham acupuncture: significant improvements were observed in the total NIH-CPSI score, pain subscore, urinary subscore, and quality of life subscore. 2. Acupuncture combined with pharmacotherapy vs pharmacotherapy alone: Demonstrated superior outcomes in the total NIH-CPSI score. 3. Optimal treatment course: 4 sessions represent the minimum effective dose, while 16 sessions achieve peak therapeutic efficacy.	Acupuncture is effective in treating CP/CPPS, with an optimal treatment course ranging from 4–16 sessions. The core acupoints include SP6, CV4, CV3, BL32, and SP9.
Liu et al^[41]^	A total of 10 RCTs, comprising 754 patients with CP/CPPS, were included.	Acupuncture (manual/electroacupuncture/catgut embedding)	1. Sham acupuncture 2. Pharmacotherapy alone (e.g., alpha-blockers, antibiotics)	1. Acupuncture vs sham acupuncture: Significant improvements were observed in the total NIH-CPSI score and response rate. 2. Acupuncture vs pharmacotherapy: Demonstrated superior pain subscore improvement, though slightly inferior urinary subscore outcomes compared to pharmacotherapy. 3. Laboratory findings: A reduction in prostatic fluid IL-1β levels was observed following acupuncture treatment.	Ac upuncture is effective in treating CP/CPPS, demonstrating significant advantages in pain relief, whereas pharmacotherapy shows superior efficacy in improving urinary symptoms.

CP = chronic prostatitis, CP/CPPS = chronic prostatitis/chronic pelvic pain syndrome, ESWT = extracorporeal shock wave therapy, MAs = meta-analyses, NIH-CPSI = Chronic Prostatitis Symptom Index, RCT = randomized controlled trial, SRs/MAs = systematic reviews/meta-analyses, TCM = traditional Chinese medicine.

Regarding the mechanisms of acupuncture for CP/CPPS, Lee et al investigated acupuncture’s impact on the immune system in CP patients. They observed significantly higher natural killer cell levels in acupuncture-treated patients compared to those receiving sham acupuncture. The anti-inflammatory and analgesic effects of acupuncture were also demonstrated in rat models of CP, though the underlying mechanisms were not further elucidated.^[42,43]^ Electroacupuncture primarily modulates the immune system by participating in the regulation of leukocyte, neutrophil, and cytokine handling processes, thereby exerting anti-inflammatory and analgesic effects.^[44]^ Furthermore, Xu et al demonstrated in rat models that electroacupuncture exerts anti-inflammatory and analgesic effects by inhibiting the release of pro-inflammatory mediators in CPPS rats, achieved through blockade of P2X7R/NLRP3 signaling pathway activation.^[45]^ Moreover, Li et al applied electroacupuncture to CP/CPPS rat models, observing attenuated pelvic pain and inflammatory responses. They proposed that this therapeutic effect may be mediated through suppression of the TLR4/NF-κB signaling pathway.^[46]^Subsequently, Hu et al experimentally verified in rat models that electroacupuncture treatment suppresses the TLR4/NF-κB signaling pathway.^[47]^ Over the past few decades, numerous clinical trials have been conducted to assess the clinical efficacy of acupuncture in treating CP. Table 8 summarizes the clinical trial data related to acupuncture that were included in this article.

**Table 8:** The literature materials included in this article and the commonly used acupuncture points.

Author/year	Intervention target	Intervention methods	Acupoints
Lee et al^[16]^	39 patients with CP/CPPS	Electroacupuncture	Bilateral BL32 (Ciliao), BL33 (Zhongliao), and GB30 (Huantiao)
Lee et al^[17]^	12 patients with CP/CPPS	Acupuncture	CV4 (Guanyuan)CV1 (Huiyin), SP6 (Sanyinjia), and SP9
Lee et al^[18,28]^	90 patients with CP/CPPS	Acupuncture	CV4 (Guanyuan)CV1 (Huiyin), SP6 (Sanyinjia), and SP9 (Yinlingquan)
Qin et al^[19]^	68 patients with CP/CPPS	Acupuncture	Bilateral BL33 (Zhongliao), BL23 (Shenshu), BL35 (Huiyang), and SP6 (Sanyinjiao)
Sahin et al^[20]^	100 male patients with type IIIB CP–CPPS	Acupuncture	BL33 (Zhongliao), BL-34 (Xialiao), BL-54 (Zhibian), CV1 (Huiyin), CV4 (Guanyuan), SP6 (Sanyinjiao), and SP9 (Yinlingquan)
Tugcu et al^[21]^	97 patients with CP/CPPS	Acupuncture	Bilateral BL33 (Zhongliao)
Sun et al^[22]^	440 patients with moderate to severe CP/CPPS	Acupuncture	Bilateral BL33 (Zhongliao), BL35 (Huiyang), BL23 (Shenshu), and SP6 (Sanyinjiao)
Küçük et al^[23]^	54 male patients with type IIIB CP–CPPS	Acupuncture	UB28 (Back-shu), GB41 (Tsu lin ch’i), LIV3 (Taichong), LI4 (Guanyu), SP6 (Sanyinjia), and SP8 (Diji)
Zhou et al^[24]^	77 patients with CP/CPPS	long-needle acupuncture	Bilateral BL30 (Baihuan Shu) and BL35 (Huiyang)
Zhou et al^[27]^	60 patients with CP/CPPS	Acupuncture	Bilateral BL23 (Shenshu), BL33 (Zhongliao), BL35 (Huiyang), and SP6 (Sanyinjiao)
Wang et al^[31]^	166 patients with CP/CPPS	Acupuncture combined with Tamsulosin	BL23 (Shenshu), BL33 (Zhongliao), and SP6 (Sanyinjiao)
Lei et al^[32]^	A certain number of CP patients (the exact quantity is unknown) who meet the specific diagnostic criteria and have been excluded from the list	Acupuncture combined with Tamsulosin	Bilateral BL33 (Zhongliao), BL35 (Huiyang), BL23 (Shenshu), and SP6 (Sanyinjiao)
Xu et al^[44,45]^	36 male Sprague–Dawley rats	Electroacupuncture	CV4 (Guanyuan), CV3 (Zhongji), Bilateral SP6 (Sanyinjiao), and Bilateral BL35 (Huiyang)
Li et al^[46]^	27 male Sprague–Dawley rats	Electroacupuncture	CV3 (Zhongji), CV4 (Guanyuan), and Bilateral KI12 (Dahe)
Hu et al^[47]^	45 male Sprague–Dawley rats	Electroacupuncture	BL30 (Baihuan Shu) and BL35 (Huiyang)

CP = chronic prostatitis, CP/CPPS = chronic prostatitis/chronic pelvic pain syndrome.

### 3.2. Chinese herbal formula

Chinese herbal formulas represent one of the most fundamental therapeutic modalities in TCM, with extensive clinical applications across diverse pathologies. CP ranks among the most prevalent prostatic disorders in clinical practice. Its management primarily focuses on pain alleviation, improvement of voiding symptoms, and enhancement of quality of life. However, while conventional interventions such as antibiotics and nonsteroidal anti-inflammatory drugs may provide symptomatic relief, they necessitate chronic administration and are associated with significant adverse effects and frequent recurrence rates.^[48]^ TCM exhibits therapeutic efficacy against this disease; however, insufficient experimental research has been conducted, and the therapeutic mechanism remains unclear. This hinders the widespread application of TCM.^[49]^ In the RCT conducted by Zhang et al, the Aike Mixture significantly reduced inflammatory cell infiltration in prostatitis; however, it demonstrated no significant effect on fibroblast proliferation.^[50]^ Liu et al postulated that symptoms of CP/CPPS may be partially attributable to compromised blood circulation. Consequently, they administered a traditional blood-activating formula for intervention. Following treatment, significant reductions were observed in serum PGE2 levels in the rat model group, accompanied by downregulated cyclooxygenase-2 (COX-2) expression in both the prostate and spinal dorsal horn. Their preliminary findings suggest that enhancing blood circulation may suppress COX-2 levels in the spinal cord, thereby exerting analgesic effects.^[51]^ Zhang et al demonstrated that Qianyu Decoction and its extracts (total flavonoids, total polysaccharides, and total saponins) exert both prophylactic and therapeutic effects on CP/CPPS, with marked synergistic effects observed among the constituent extracts.^[52]^ Xiong et al investigated the effects of Bazheng San on CNP.^[53]^ Results demonstrated that Bazheng San significantly reduced levels of interleukin (IL)-6, IL-8, and IL-17, while elevating secretory immunoglobulin A (SIgA) levels. It concurrently decreased monocyte chemoattractant protein-1 (MCP-1), vascular cell adhesion molecule-1, and fibroblast growth factor-2. Notably, substantial reductions were observed in levels of very late antigen-4, C-C motif chemokine ligand 2, and fibroblast growth factor receptor-1. The formulation markedly suppressed inflammatory cell infiltration, stromal hyperplasia, and tissue destruction in the prostate, reduced collagen deposition in the stromal region, decreased prostate fluid leukocyte count, and increased lecithin body density. These effects exhibited dose-dependent efficacy, with the high-dose group (100 mg/kg) demonstrating particularly pronounced outcomes. Subsequently, Xiong et al developed Modified Bazheng San (mBazheng San) by replacing *Gardenia jasminoides* and *Rheum palmatum* in the original Bazheng San decoction with *Pteris multifida* and *Evodia rutaecarpa*. This modified formulation demonstrated enhanced efficacy in ameliorating inflammatory cell infiltration and stromal hyperplasia, suppressing bacterial growth in prostatic tissue, achieving significantly greater reductions in IL-1β, IL-8, and tumor necrosis factor-α (TNF-α) levels, elevating SIgA levels, and more effectively inhibiting collagen deposition in the prostatic stroma.^[54]^ In a RCT conducted by Cui et al investigating Qianlie Tongli Decoction, the high-dose group (1.0 g/mL) significantly alleviated pain in mice, markedly reduced inflammatory cell infiltration, and substantially decreased serum TNF-α levels.^[55]^

### 3.3. Botanical preparations

Botanical preparations is widely employed as an alternative therapeutic approach for CP/CPPS with varying degrees of clinical efficacy, particularly utilizing pollen extract, quercetin, or saw palmetto.^[56]^ Cernilton, a specific pollen extract, has demonstrated efficacy against multiple urological disorders.^[57]^ Its active components consist of a water-soluble fraction (T-60) and a fat-soluble fraction (GBX).^[58]^ As early as 1989, Buck et al demonstrated that Cernilton exhibits potent anti-inflammatory properties capable of alleviating pain symptoms in CP/CPPS patients.^[59]^ Subsequently, a prospective study by Rugendorff et al administered Cernilton to CP patients at a dosage of 1 tablet 3 times daily (tid) for 6 months, demonstrating favorable responses in 78% of patients and symptomatic resolution in 36% of cases.^[60]^ Subsequently, an experimental study by Kmijo et al utilizing a rat model demonstrated through histopathological analysis of prostate tissue (including glandular damage, cellular proliferation, and apoptosis) that Cernilton predominantly protects acinar epithelial cells via its GBX fraction and suppresses stromal hyperplasia primarily through enhanced apoptosis mediated by the T-60 fraction. In a RCT by Cai et al, the combination of Cernilton with B-complex vitamins (B_1_, B_2_, B_6_, B_9_, B_12_, and PP [niacin]) demonstrated superior symptomatic improvement and more significant reduction in serum IL-8 levels compared to monotherapy.^[61]^ An in vitro study by Dizeyi et al further demonstrated that Cernilton upregulates the anti-inflammatory cytokine IL-10 and enhances TNF-related apoptosis-inducing ligand levels in human peripheral blood mononuclear cells, while significantly reducing androgen receptor and prostate-specific antigen protein expression in stromal cells.^[62]^ A prior multicenter, randomized, prospective, double-blind, placebo-controlled phase 3 trial by Wagenlehner et al demonstrated significant improvements in NIH-CPSI scores and QoL among inflammatory CP/CPPS patients, with no severe adverse events reported. In a recent randomized, controlled, single-blind, phase III study by Cai et al, the combination of Cernilton with hyaluronic acid and vitamins demonstrated superior improvement in patients’ NIH-CPSI scores and QoL compared to ibuprofen, alongside a significant reduction in white blood cell counts.^[63]^

Quercetin is a polyphenolic bioflavonoid naturally occurring in fruits, vegetables, and grains.^[64]^ It has been demonstrated to possess antioxidant and anti-inflammatory properties,^[65]^ and exerts anticancer effects through multiple mechanisms including inducing apoptosis and inhibiting cell proliferation.^[66]^ In a prospective, randomized, double-blind, placebo-controlled trial by Shoskes et al involving patients with Category III CP syndrome, 67% of subjects demonstrated significant symptomatic improvement following quercetin monotherapy. Subsequently, 82% of patients in a separate cohort exhibited marked symptom relief after treatment with Prosta-Q (a formulation containing quercetin, bromelain, and papain – enzymes enhancing quercetin bioavailability), with a favorable tolerability profile observed.^[67]^ Meng et al established a novel CP/CPPS rat model using complete Freund adjuvant. Administration of low- (50 mg/kg/d), medium- (100 mg/kg/d), and high-dose (200 mg/kg/d) quercetin significantly reduced prostate index, inflammatory cell infiltration, lymphocyte infiltration (CD3⁺ T cells and CD19⁺ B cells), and tissue levels of pro-inflammatory cytokines (IL-1β, IL-2, IL-6, IL-17A, MCP-1, and TNF-α). Concurrently, it enhanced antioxidant capacity in CP/CPPS rats by increasing antioxidant enzymes and reducing lipid peroxidation products. Preliminary mechanistic exploration indicated that quercetin’s anti-inflammatory and antioxidant activities are partially mediated through downregulation of the NF-κB and MAPKs signaling pathway.^[68]^

Saw palmetto is another phytotherapeutic agent that alleviates lower urinary tract symptoms associated with benign prostatic hyperplasia. Its proposed mechanisms include inhibition of lipoxygenase and COX, reduction of testosterone conversion to dihydrotestosterone, and blockade of α1-adrenergic receptors.^[69]^ In a prospective, randomized, open-label study comparing saw palmetto with finasteride, saw palmetto demonstrated no significant symptomatic improvement in CP/CPPS patients, suggesting its therapeutic efficacy remains indeterminate.^[70]^

### 3.4. Tuina

Within the theory and practice of TCM, appropriate Tuina massage is recognized for improving local blood circulation and alleviating muscle tension, demonstrating adjunctive efficacy in mitigating symptoms of CP/CPPS. A study by Shoskes et al substantiated that prostate massage combined with specific antibiotics exhibits therapeutic benefits for certain patients with long-term refractory CP.^[71]^ In contrast, a study by Ateya comparing antibiotic monotherapy versus combination therapy with prostate massage and specific antibiotics yielded discrepant findings. The adjunctive prostate massage failed to demonstrate significant enhancement of therapeutic outcomes in the refractory cohort.^[72]^ A RCT by Ying et al comparing perineal essential oil massage versus placebo demonstrated that while the massage showed no significant effect on patients’ total NIH-CPSI scores, pain sub-scores, or quality of life sub-scores, it significantly alleviated perineal discomfort and localized pain.^[73]^ In a recent study by Wu et al, a combined therapy of Traditional Chinese Herbal Medicine Retention Enema and perineal massage targeting the Qichong (ST30) and Chongmen (SP12) acupoints was applied to patients with refractory CP/CPPS. The results demonstrated significant reductions in Visual Analog Scale pain scores, International Prostate Symptom Scores, NIH-CPSI scores, and QoL scores. Subjective symptom relief was substantially alleviated, confirming the marked therapeutic efficacy of this strategy for refractory CP/CPPS.^[74]^

## 4. Discussion

CP/CPPS represents a prevalent disorder in the male genitourinary system, with an incidence rate ranging from 4.5% to 9.0% among young males and recurrence rates reaching up to 50% in older male populations.^[75]^ In this review, we initiated a bibliometric analysis to evaluate current developmental trends and future research directions in the field. As previously noted, recent years have witnessed growing emphasis on TCM research for CP/CPPS management, particularly from Chinese institutions and scholars. Leading organizations such as Beijing University of Chinese Medicine and China Academy of Chinese Medical Sciences demonstrate dominant publication output and citation impact, underscoring China’s substantial commitment to this domain. Our analysis further revealed that Medicine is the journal publishing the highest volume of articles in this field, indicating recognition of TCM among pharmacology experts. Furthermore, keyword analysis identified acupuncture as the primary TCM modality for treating CP/CPPS, including approaches such as traditional acupuncture, electroacupuncture, long-needle therapy, trigger point injection, minimally invasive acupuncture and Acupuncture Combined with Western Medication. Analysis of burst keywords revealed that current research hotspots in this field primarily focus on oxidative stress and CNP. This finding will guide our future research direction, emphasizing the relationship between CNP and oxidative stress mechanisms. Subsequently, we conducted a SR of research progress on various TCM modalities (including acupuncture, herbal decoctions, Tuina massage, and phytoformulations) for managing CP/CPPS. This review comprehensively discusses recent advances in TCM interventions for CP/CPPS and their underlying mechanisms, providing valuable references for clinical practice.

### 4.1. Bibliometric analysis

Based on the bibliometric analysis presented in this paper, it is observed that research on TCM for the treatment of CP/CPPS has experienced significant growth in both publication and citation volumes since 2011. Although a slight decline has been noted in recent years, output has remained stable. In terms of international collaboration, China serves as the central hub of research activity, while contributions from other countries remain limited, and cooperation among them is sparse. Future efforts should prioritize promoting China-centered global collaborative initiatives. Furthermore, it is noteworthy that high-quality studies in this field are still scarce, and investigations into therapeutic mechanisms remain at a nascent stage, with no clearly defined signaling pathways identified to date. Moving forward, research should focus on elucidating the mechanisms underlying TCM interventions for CP/CPPS through in-depth studies, producing high-quality publications, and enhancing the global influence of TCM.

### 4.2. Efficacy and mechanism analysis

Acupuncture is a therapeutic method that involves inserting needles into specific acupoints to regulate bodily functions. It is currently among the most promising and extensively studied interventions within TCM for managing CP/CPPS. The commonly used acupoints are shown in Table 8. Stimulation of these acupoints alleviates CP/CPPS symptoms and improves quality of life through mechanisms such as anti-inflammatory effects, analgesia, and improved voiding dysfunction. This modality demonstrates high safety, low cost, technical simplicity, and favorable outcomes. The underlying therapeutic mechanisms primarily involve immunomodulation via regulation of inflammatory cytokines and suppression of immune responses, specifically through modulation of TLR4 and NF-κB expression levels. This effect is further mediated by inhibiting activation of the P2X7R/NLRP3 signaling pathway. This is achieved through downregulation of key pro-inflammatory mediators, including reduced concentration and bioactivity of IL-1, IL-6, IL-8, and TNF-α. Moreover, it reduces the level of PGE2 by decreasing the expression of COX-2. Furthermore, acupuncture modulates immune cell dynamics by enhancing natural killer cell activity while reducing leukocyte and neutrophil infiltration, thereby attenuating prostatic inflammation. Furthermore, acupuncture exerts analgesic effects by modulating neurotransmitter release, with clinical studies demonstrating elevated concentrations of β-endorphin and leucine–enkephalin following treatment. Regarding voiding dysfunction improvement, acupuncture normalizes urethral sphincter tone, thereby enhancing maximum urinary flow rate (*Q*_max_) and mean urinary flow rate (*Q*_ave_).^[76]^ Although numerous studies have demonstrated the efficacy of acupuncture in treating CP/CPPS with a favorable safety profile, several methodological challenges remain. These include inconsistent selection of acupoints across studies, inability to identify the primary active points, and a lack of evidence regarding potential synergistic effects among multiple acupoints. Furthermore, there is considerable heterogeneity in needling techniques, with limited comparative effectiveness research among different approaches. The optimal treatment frequency, session duration, and overall course of acupuncture have not been firmly established. Although various regimens have shown some therapeutic benefits, robust evidence-based guidelines are still lacking. Future research should focus on identifying the most effective acupoint combinations, needling techniques, treatment parameters, and session protocols. Additionally, the depth of needle insertion for each acupoint should be standardized to maximize treatment efficacy while minimizing the number of sessions required.

As the predominant modality in TCM therapy, herbal decoctions demonstrate extensive clinical applicability. Numerous herbs possess therapeutic properties including heat-clearing and detoxification, blood-activating and stasis-resolving effects, along with antibacterial and anti-inflammatory actions. These effectively alleviate CP/CPPS symptoms such as urinary frequency, urgency, and dysuria. Mechanistic studies indicate that TCM formulations stimulate systemic circulation, suppress COX-2 expression leading to significantly reduced PGE₂ biosynthesis, downregulate pro-inflammatory mediators (IL-1β, IL-6, IL-8, IL-17, TNF-α, MCP-1, vascular cell adhesion molecule-1, fibroblast growth factor-2), and enhance SIgA levels in mucosal immunity. These effects collectively inhibit inflammatory cell infiltration, attenuate tissue hyperplasia and destruction, reduce collagen deposition, and have demonstrated significant alleviation of pelvic pain and improvement in lower urinary tract symptoms among patients.

Pollen extract, as a natural mixture, significantly improves symptoms in patients with CP/CPPS, with no significant adverse events. The primary mechanism for symptom alleviation lies in its potent anti-inflammatory properties and antioxidant effects. The anti-inflammatory action is primarily achieved by inhibiting nitric oxide production and COX-2 activity, reducing the levels of pro-inflammatory cytokines (such as IL-1β, IL-2, IL-6, IL-17A, MCP-1, and TNF-α), decreasing inflammatory cell and lymphocyte infiltration, and elevating the level of the anti-inflammatory cytokine IL-10. The antioxidant effects are primarily mediated by inhibiting 5α-reductase activity, thereby suppressing the conversion of testosterone to dihydrotestosterone.^[77]^ These anti-inflammatory and antioxidant properties are potentially mediated through downregulation of the NF-κB and MAPKs signaling pathways. Furthermore, it exhibits anti-proliferative effects by protecting glandular epithelial cells and enhancing cellular apoptosis, thereby suppressing prostatic stromal hyperplasia. Chinese herbal formulations and pollen extracts serve as alternative therapeutic approaches within TCM for managing CP/CPPS, showing promising symptomatic relief. However, most existing studies remain largely confined to basic research, with a notable lack of well-designed clinical trials (both multicenter and single-center) to further validate their efficacy. Future efforts should prioritize the clinical translation of herbal decoctions and pollen extracts to provide patients with additional treatment options.

Tuina massage, a common modality in TCM, improves local blood circulation and alleviates muscle tension. For CP/CPPS patients, prostatic massage or perineal massage serves as an adjunctive therapy. In patients refractory to pharmacological and other conventional treatments, massage facilitates the expulsion of obstructive material from prostatic ducts and enhances peripheral prostatic circulation, thereby reducing pain.

TCM holds significant clinical value in prostatitis treatment, with its core significance lying in addressing the therapeutic gap of targeted therapies in Western medicine. Conventional Western approaches to CP/CPPS primarily rely on antibiotics and alpha-blockers. However, these conventional treatments exhibit limited efficacy and high recurrence rates. In contrast, TCM exerts comprehensive therapeutic effects through multi-target regulatory mechanisms, it not only significantly downregulates pro-inflammatory cytokine expression and mitigates tissue inflammation, but also modulates pain-associated pathways such as COX-2/PGE2 to alleviate pain. Therapeutically, TCM can reduce antibiotic dependency and lower drug resistance risks. Clinical studies demonstrate that when herbal formulations like Ningmitai capsules are combined with antibiotics, they not only demonstrate superior improvement in patients’ pain symptoms and quality of life but also avoid alpha-blocker-associated adverse effects such as dizziness and orthostatic hypotension.^[78]^ Collectively, TCM not merely alleviates localized symptoms but also enhances quality of life through systemic modulation, thereby delivering an enhanced therapeutic approach for prostatitis. Although TCM has demonstrated promising outcomes in CP/CPPS management, significant research opportunities remain. Future investigations should prioritize elucidating the mechanistic basis of TCM interventions through advanced methodologies such as molecular biology and metabolomics. Such research should examine how herbal formulations and acupoint stimulation modulate systemic responses to alleviate pain, improve voiding dysfunction, and address sexual dysfunction. Concurrently, the field requires high-quality, large-scale, multicenter RCTs to validate the efficacy and safety of TCM therapies, thereby strengthening the evidence base for clinical implementation. Furthermore, optimizing precision-based individualized treatment strategies that account for patient-specific variables including constitution, disease severity, and chronicity will enhance clinical efficacy. Finally, advancing integrated Chinese–Western medicine research to identify synergistic therapeutic models will provide comprehensive treatment paradigms, facilitating broader global adoption of TCM approaches in CP/CPPS care.

However, this SR has several inherent limitations. First, the exclusive reliance on publications from the WOSCC may have resulted in incomplete literature retrieval. Nevertheless, WOSCC’s rigorous selection criteria and comprehensive coverage of high-impact journals ensure it accurately represents the current research landscape in this field. Second, only English-language publications were included. While this review aims to elucidate TCM therapeutic strategies and their underlying mechanisms for CP/CPPS management where interventions such as herbal formulations and acupuncture have demonstrated significant clinical efficacy their precise mechanisms of action remain incompletely characterized and warrant further mechanistic investigation.

## 5. Conclusion

TCM has accumulated substantial empirical experience and achieved notable progress in managing CP, with increasing international collaboration advancing this field. Modalities including herbal medicine, acupuncture, and Tuina massage demonstrate clinically relevant efficacy in alleviating symptoms and improving QoL for CP patients. Their potential mechanisms may involve immunomodulation, enhanced local circulation, attenuated inflammatory responses, and neuroendocrine system regulation. However, therapeutic outcomes exhibit considerable interindividual variability due to the heterogeneous nature of CP. Consequently, treatment selection requires comprehensive evaluation of patient-specific factors under specialist guidance, employing integrated therapeutic approaches. Future clinical investigations should further elucidate TCM’s mechanistic pathways and therapeutic outcomes to optimize clinical implementation.

## Author contributions

**Conceptualization:** Debo Li.

**Data curation:** Debo Li.

**Formal analysis:** Debo Li, Yongfeng Lao, Xin Guan.

**Visualization:** Debo Li, Yongfeng Lao, Xin Guan.

**Writing – original draft:** Debo Li, Yongfeng Lao, Xin Guan.

**Writing – review & editing:** Xiangbin Kong, Zhilong Dong.
